# Prevalence of obsessive-compulsive disorder in Turkish university students and assessment of associated factors

**DOI:** 10.1186/1471-244X-9-40

**Published:** 2009-07-06

**Authors:** Elcin Yoldascan, Yarkin Ozenli, Oguz Kutlu, Kenan Topal, Ali Ihsan Bozkurt

**Affiliations:** 1 Department of Public Health, Faculty of Medicine, Cukurova University, Adana, Turkey; 2 Department of Psychiatry, Faculty of Medicine, Baskent University, Ankara, Turkey; 3 Department of Computer and Teaching Technology Education, Faculty of Education, Cukurova University, Adana, Turkey; 4 Department of Family Medicine, Faculty of Medicine, Pamukkale University, Denizli, Turkey; 5 Department of Public Health, Faculty of Medicine, Pamukkale University, Denizli, Turkey

## Abstract

**Background:**

Many students who begin university at risky periods for OCD development cannot meet the new challenges successfully. They often seek help and apply to the university health center for psychiatric distress. We aimed to determine the prevalence and associated factors of Obsessive Compulsive Disorder (OCD) at students of the Cukurova University in this cross sectional study.

**Methods:**

This study was performed in the Cukurova University Faculty of Education with a population of 5500 students; the representative sample size for detecting the OCD prevalence was calculated to be 800. After collecting sociodemographic data, we questioned the students for associated factors of OCD. The General Health Questionnaire-12 (GHQ-12) and Composite International Diagnostic Interview (CIDI, Section K) were used for psychiatric evaluation. Logistic regression analysis was performed to evaluate the linkage between OCD and associated factors.

**Results:**

A total of 804 university students were included in this study. The GHQ-12-positive students (241 students, 29.9%) were interviewed using Section K of the CIDI (222 students, 27.6%). OCD was diagnosed in 33 (4.2%) students. The Logistic regression analysis of the data showed significant associations between OCD and male gender (p:0.036), living on government dormitory (p: 0.003), living on students' house/parental house (p:0.006), having private room in the parental house (p:0.055) and verbal abuse in the family (p:0.006).

**Conclusion:**

This study demonstrates a higher prevalence of OCD among a group of university students compared to other prevalence studies of OCD in Turkish society. Furthermore, our findings also suggest relationships between OCD and sociodemographic factors, as well as other environmental stress factors.

## Background

Obsession is defined as an unwanted, intrusive, improper, recurrent, and continual thought, impulse, and/or mental image. Compulsion refers to repetitious behavioral and/or mental activities. Obsessions are usually perceived to be excessive and senseless by the external world and often cause considerable distress to their sufferers. Obsessive-compulsive events usually consume at least an hour of the sufferer's daytime period and cause embarrassment, especially in social, occupational, and other daily situations [1]. Studies investigating the lifelong prevalence of Obsessive-Compulsive Disorder (OCD) reveal concordant results. The prevalence ranges between 1.3% and 5.5%, and OCD presents itself in 2.7% of the general population [1,2]. OCD is categorized under the group of anxiety disorders in DSM IV. Although other anxiety disorders in this category occur more frequently in men than women (female/male: 2/1), the ratio of female/male prevalence is equal for OCD [3,4]. Genetics, temperament, stressful life events, and modeling parental behavior are all implicated in the etiology of the disorder. Clinical obsessions include the fear of dirt/germs, a yearning for symmetry/certainty, suspicion, sexuality, and a fixation on religion. Thus, compulsions often include rituals focused on cleansing, controlling, arranging, counting, touching, and collecting [5].

Although the age of onset varies, the most risky periods for OCD development are adolescence and young adulthood [4,6]. When they begin their new life in the university, those who cannot meet the new challenges successfully often seek help and apply to the university health center for psychiatric distress. They usually experience feelings of distress and hopelessness. These sentiments can translate into clinical depression, general anxiety, interpersonal relationship issues, behavioral disorders, and OCD [7,8]. However, very few studies in the literature address OCD among university students [9,10]. Moreover, there are no methodical surveys that investigate the epidemiology of OCD in university students.

Therefore, the goal of this study is to determine the lifelong prevalence and accompanying factors for OCD among university students. Even though the sample used is limited in its scope, we hope that this epidemiological study can serve as the basis for future cross-cultural comparisons.

## Methods

### Subjects and Study Design

This cross-sectional epidemiological study was conducted in the Cukurova University Faculty of Education Approval of the Ethics Committee of Cukurova University was obtained. A total of 5500 students were included in the study, and the representative sample size for OCD prevalence detection was calculated to be 800 (α: 0.05, p: 2.5% and d: 2%). The study had two phases and was carried out from July 2006 to July 2007, with a maximum interval of 15 days, to avoid any changes in mental state. The first phase involved the application of a sociodemographic data form, which also included questions about environmental conditions. We randomly selected one of the nine departments of the Faculty of Education and visited this department during the first two days of the week. All of the students who attended class on these days were included in the study. The 12-item General Health Questionnaire (GHQ-12) was used to screen for psychiatric morbidity especially in primary care. There is evidence that the GHQ correlates well with other psychiatric screening tests. [11,12] The validity and reliability of the Turkish version of the GHQ was previously approved. The reliability correlation, sensitivity, and specificity of the GHQ in this study were 0.78, 0.74, and 0.84, respectively. [13,14] GHQ-12-positive students were selected for the second phase and invited to the university health center. The students who respond to this invitation were interviewed using Section K of the CIDI (OCD K1–K21, Obsessive Compulsive Disorders Interview Criterion). The interviews administered by a public health specialist, by a psychiatrist and the general practitioners who were trained for CIDI and working in university health center. A qualitative assessment was subsequently performed by a psychiatrist to confirm the presence of OCD according to DSM IV criteria. [15,16]

### Instrument

#### General Health Questionnaire (GHQ-12)

It is a self administered screening test for detecting poor mental health in the general population. This questionnaire has been widely used in many countries for detecting psychological morbidity since its development by Goldberg in 1970, subjects are asked to think about their health over the past few weeks and answer the questions accordingly. There were four response options for each item (better than usual, same as usual, less than usual, much less than usual). We used a bimodal response scale known as GHQ scoring; columns 1 and 2 are both scores 0, and columns 3 and 4 are both scored 1. This bimodal response scale is a simple method of scoring and eliminates errors due to "end-users" and "middle-users". [17] We take a cut off point 1/2 (maximum score 12) for indicating poorer psychological health. [18]

#### Sociodemographic data form

Part I consisted of personal data, such as age, sex, marital status, socioeconomic level, residential place, illness history, surgical history, and psychiatric treatment history. Part II included questions about family members, such as the number of rooms in the parental house, private room in the parental house, number of siblings, number of households, parental educational and socioeconomic levels, and history of verbal/physical abuse in the family. The answers of the students responded the items for verbal abuse were about belittling, screaming, threats, blaming or sarcasm in the family. And the items responded for physical abuse were about any act resulting with non accidental physical injury like beating, punching, biting and kicking and exposure to unreasonably severe corporal punishment or unjustifiable punishment in the family. [19]

#### Composite International Diagnostic Interview (CIDI 2.1)

This interview was developed by the World Health Organization. It is a comprehensive and fully-standardized diagnostic interview designed to assess mental disorders according to the definitions provided by the ICD-10 Diagnostic Criteria for Research and DSM IV [13]. It is composed of three parts: Part I include logical questions; Part II includes subject-oriented questions; Part III includes criterion-based questions. Psychiatric disorders can be diagnosed through "yes" or "no" answers to the questions in each diagnostic section. Responses are then evaluated according to a five-point scale: level 1) mental illness is not present; level 2) mental illness is present but not critical; level 3) mental illness is dependent on drug or substance abuse; level 4) mental illness is dependent on physical illness or injury; level 5) mental illness is present, and the cause is psychological. These scores can be converted into psychiatric diagnoses via specialized software. The CIDI can be applied by non-medical personnel after training. This interview requires approximately 70 minutes under normal circumstances [20].

### Statistical Analyzes

After descriptive statistics, were obtained the presence of OCD and relationship of the independent variables were analyzed by binary assessments. A Chi-square test was used for analyses. Then, logistic regression analysis (LRA) was performed to analyze the effect of these variables together. Before the LRA, the correlation coefficient between independent variables was calculated. According to these calculations, there was a high correlation between students' own economic situation and both parental economic situation as well as the education level of the father and mother (r: 0.70 and r: 0.61 respectively). Therefore, only students' own economic situation and the education level of the mother were included in the model.

The independent variables included in the LRA model were the class and department that the student was attending in the Faculty of Education, gender, marital status, students' own economic situation, residential place, number of siblings, number of households, number of rooms in the parental house, presence of a private room in the parental house, history of chronic illness, operation history, verbal and physical abuse in the family, history of verbal and physical abuse, and education level of the mother.

## Results

A total of 804 students were included in this study. The GHQ-12-positive subjects (241 students, 29.9%) were invited to the university health center. The students who responded (222 students, 27.6%) were interviewed using Section K of the CIDI. OCD was diagnosed in 33 students and we found the prevalence of OCD (4.2%) after excluding the nineteen students who did not respond to our invitation (Table 1). The non responding students have various reasons; eight had a physical illness, five had gone other universities, two drop out school, one had gone abroad and three of them reject to participate to the study.

**Table 1:** Total population and results of screening.

Total population of the Cukurova University Faculty of Education students	5500
The students screened with the GHQ-12	804

GHQ-12 positives	241 (29.9%)

Students who did not respond for various reasons	19 (2.6%)

Interviewed with Section K of the CIDI	222 (27.6%)

Students who screened and interviewed	785*

Prevalence of OCD	33 (4.2%)

*Students who did not interviewed were excluded.

The students' sociodemographic features are listed in Table 2. From the subjects, 510 (63.5%) were female and 294 (36.5%) were male. The parents of 288 (35.8%) of the participants lived in Adana (the city in which the university is located); the remaining students' parents (64.2%) dwelled in other Turkish cities. The education levels of the students' mothers were as follows: 160 (19.9%) were illiterate; 59 (7.3%) were literate (i.e. they could read and write but had no formal education); 342 (42.5%) were primary school graduates; 150 (18.7%) were middle school graduates; 86 (10.7%) were high school or university graduates.

**Table 2:** Sociodemographic and characteristic features of study group and students diagnosed OCD.

**Sociodemographic and characteristic features**		**Study Group** **n = 804**		**OCD Group** **n = 33**	
		
		**n**	**%**	**n**	**%**
Gender	Male	293	36.5	9	27.3
	
	Female	510	63.5	24	72.7

Marital Status	Single	781	97.6	31	93.9
	
	Married	19	2.4	2	6.1

Economic Situation	Poor	127	15.8	3	9.1
	
	Middle	606	75.4	29	87.9
	
	Good	68	8.5	1	3.0

Residential Place	Government dormitory	276	32.1	9	27.3
	
	Private dormitory	26	3.2	1	3.0
	
	Students' house/parental house	499	62.3	20	60.6
	
	Other	19	2.4	3	9.1

Education level mother	Illiterate	160	20.1	10	30.3
	
	Literate	59	7.4	-	-
	
	Primary school	342	42.9	11	33.3
	
	Middle school	150	18.7	8	24.2
	
	Higher Education	86	10.7	4	12.1

Private room in the parental house	Exist	448	55.7	24	72.7
	
	Not exist	356	44.3	9	27.3

Verbal abuse in the family,	Exist	55	6.9	8	24.2
	
	Not exist	745	93.1	25	75.8

Physical abuse in the family	Exist	21	2.6	2	6.1
	
	Not exist	781	97.4	31	93.9

Own history of verbal abuse	Exist	114	14.2	2	6.1
	
	Not exist	689	85.7	31	93.9

Own history of physical abuse	Exist	5	0.6	-	-
	
	Not exist	799	99.4	33	100.0

Our results suggest relationships between OCD and female gender, living on government dormitory, living on students' house/parental house, having private room in the parental house and verbal abuse in the family (OR = 0.262, p = 0.036; OR = 0.035, p = 0.003; OR = 0.054, p = 0.006; OR = 2.795, p = 0.055; OR = 9.203, p = 0.006 respectively) (Table 3).

**Table 3:** The relationship between the independent variables and OCD.

**Independent variables**	**p**	**OR**	**95.0% CI for OR**	
			
			**Lower**	**Upper**
**The Department Of The Student**	0.977			

Art Teaching	0.897	10842.1	0.001	1.714

Primary School Teacher	0.882	41953.6	0.001	6.949

Computer And Teaching Technology	0.878	63265.1	0.001	1.033

Early Childhood Education	0.997	1.5	0.001	1.403

Philosophy Group	0.886	29958.1	0.001	4.720

German Language Teaching	0.982	22.1	0.001	1.154

French Language Teaching	0.991	3.269	0.001	2.448

English Language Teaching	0.884	37112.6	0.001	6.153

Turkish Language Teaching	0.890	21523.8	0.001	3.413

Science Knowledge	0.867	170746.5	0.001	2.885

Social Sciences	0.869	139598.1	0.001	2.333

Psychological Counseling And Guidance	0.878	60296.3	0.001	9.806

**The Class Of The Student**	0.654			

Class 1	0.287	7.390	0.186	29.391

Class 2	0.623	2.015	0.123	32.964

Class 3	0.457	1.864	0.361	9.625

**Male Gender**	**0.036**	0.262	0.075	0.917

**Being Single**	0.086	0.167	0.021	1.291

**Economic Situation**	0.548			

Economic Situation Of The Family	0.993	0.987	0.045	21.451

Economic Situation Of The Student	0.560	2.197	0.156	30.987

**Residential Place Of Student**	0.026			

Government Dormitory	**0.003**	0.035	0.004	0.313

Private Dormitory	0.189	0.114	0.004	2.907

Students' house/parental house	**0.006**	0.054	0.007	0.435

**Number Of Siblings**	0.640	1.073	0.797	1.445

**Number Of Households**	0.142	0.798	0.590	1.078

**Private Room In The Parental House**	**0.055**	2.795	0.978	7.992

**Room Number Of The Parental House**	0.396	1.325	0.692	2.539

**Illness History**	0.796	0.001	0.001	3588

Chronic Illness History	0.066	3.565	0.918	13.850

Operation History	0.114	0.250	0.045	1.397

Mental Disorder History	0.432	2.387	0.273	20.891

**Verbal Abuse In The Family**	**0.006**	9.203	1.882	45.006

**Physical Abuse In The Family**	0.830	1.334	0.095	18.657

**Own History Of Verbal Abuse**	0.076	0.156	0.020	1.216

**Own History Physical Abuse**	0.969	0.001	0.001	5.596

**Education Level Of Mother**	0.782			

Illiterate	0.294	3.333	0.352	31.565

Literate	0.870	0.001	0.001	3158

Primary School	0.484	1.933	0.305	12.234

Middle School And Upper Level	0.265	2.869	0.450	18.307

**Constant**	0.871	0.001	0.186	293.919

Abbreviations: OR: Odds Ratio; CI: Confidence Interval

## Discussion

The observed probability of psychiatric disorder in the university student cohort used in this study was 29.9%, which is greater than the ratio found in the general Turkish population (5–20%). At this point, we should consider the role of environmental stressors as well as family systems and genetic predisposition to OCD [21]. Many students who begin university at risky periods for OCD development cannot meet the new challenges successfully. Students who experience such stressors are more likely to display tendencies towards depression, general anxiety, behavioral disorders, and somatic complaints [8,22].

We applied Section K of the CIDI to students who displayed a proclivity towards psychiatric disorders. OCD was diagnosed in 33 students (4.2% of the cohort). Although the lifelong prevalence of OCD varies widely according to the literature, the rate is 2.5% in Turkish society [23]. Adolescents and young adults experience increased physiologic and reactive anxiety symptoms, and are thus more prone to anxiety disorders. Consequently, the illness rate in this population is around 20% [1].

In preceding studies, the female/male OCD ratio was observed to be close to 1/1 [3,4]; in contrast with these findings, we observed a female/male ratio of 2.6/1. However, we have to bear in mind that anxiety disorders are generally seen two to three times more frequently in young women than young men. Also, each sex exhibits different sensitivity levels to stress and anxiety disorders. Our findings, which illustrate that women displayed a higher response to stress than men, support the results of previous epidemiological studies conducted in seven different countries [1,24]. Similarly, Horwath and Weissman concluded from their cross-national epidemiological study that the lifetime prevalence of OCD is generally higher in women than men. For example, the female-to-male ratios are consistent for Korea (1.2), Puerto Rico (1.2), Edmonton (1.3), the United States (1.6), Taiwan (1.8), and in New Zealand (4.0). [25]

Although the previous clinical studies showed a correlation between high socioeconomic status and OCD [26,27], Torres and Prince describe in their editorial comment epidemiological studies that have detected lower socioeconomic levels among OCD sufferers [28].

The relationship between childhood trauma, such as parental separation or child abuse, and anxiety disorders has been studied in recent years [29,30]. Animal studies have shown that negative experiences in childhood have a negative impact on the central nervous system and development [31,32]. Mathews et al. showed an association between emotional abuse, physical abuse and high levels of OCD symptoms in their study [33]. Lochner et al. found a significantly greater severity of childhood trauma in general and emotional neglect specifically, in the OCD groups compared to the controls [34]. We found significant positive correlations between the presence of familial verbal abuse and OCD but there was no association between physical abuse and OCD in our study. We used items for assessment verbal/physical abuse which was listed in the Diagnostic and Statistic Manual of Mental Disorders (DSM-IV-TR) under the heading of 'Other Conditions That May Be a Focus of Clinical Attention'. We thought that there can be a relation between physical abuse and OCD but our findings did not support this maybe because students display tendency not to disclose physical abuse.

There are some limitations that need to be acknowledged regarding the present study. The first limitation concerns about the method of the study. Our study was a cross-sectional epidemiological study but we know that prospective longitudinal studies are of great value for assessing psychiatric diseases. The second limitation is the co-morbid situations of the study population were not investigated and we did not ask about streptococcal infections directly although we asked about chronic illness and operation history.

## Conclusion

Our study demonstrates a higher prevalence of OCD among a group of university students compared to other prevalence studies of OCD in Turkish society. These findings also suggest relationships between OCD and sociodemographic factors, as well as other environmental stressors. More methodological and longitudinal studies are needed to determine the prevalence and associated factors for OCD in different age groups from various layers of the population.

## Competing interests

The authors declare that they have no competing interests.

## Authors' contributions

EY Conceived of the study, performed the literature review, contributed to study design and data collection, and interviewed students. YO Contributed to study design and data collection, drafted the manuscript, interviewed students and performed the qualitative assessments. OK Contributed to study design, data collection and analysis. KT Contributed to the design and coordination of the study, drafted and edited the manuscript. AIB Contributed to study design and performed the statistical analysis. All authors read and approved the final manuscript.

## Pre-publication history

The pre-publication history for this paper can be accessed here:


